# Safety and Efficacy of Hospital Utilization of Tranexamic Acid in Civilian Adult Trauma Resuscitation

**DOI:** 10.5811/westjem.2019.10.43055

**Published:** 2020-02-21

**Authors:** Michael M. Neeki, Fanglong Dong, Jake Toy, Joseph Salameh, Massoud Rabiei, Joe Powell, Richard Vara, Kenji Inaba, David Wong, Mark E. Comunale, Andrew Lowe, Deepak Chandwani, Juan Quispe, Rodney Borger

**Affiliations:** * Arrowhead Regional Medical Center, Department of Emergency Medicine, Colton, California; † California University of Science and Medicine, Colton, California; ‡ Arrowhead Regional Medical Center, Department of Surgery, Colton, California; § Arrowhead Regional Medical Center, Department of Anesthesia, Colton, California; ¶ Arrowhead Regional Medical Center, Department of Pharmacy, Colton, California; || Loma Linda University Medical Center, Department of General Surgery, Loma Linda, California; # City of Rialto Fire Department, Rialto, California; ** Univeristy of Southern California, Department of Surgery, Los Angeles, California

## Abstract

**Introduction:**

Patients with trauma-induced coagulopathies may benefit from the use of antifibrinolytic agents, such as tranexamic acid (TXA). This study evaluated the safety and efficacy of TXA in civilian adults hospitalized with traumatic hemorrhagic shock.

**Methods:**

Patients who sustained blunt or penetrating trauma with signs of hemorrhagic shock from June 2014 through July 2018 were considered for TXA treatment. A retrospective control group was formed from patients seen in the same past five years who were not administered TXA and matched based on age, gender, Injury Severity Score (ISS), and mechanism of injury (blunt vs penetrating trauma). The primary outcome of this study was mortality measured at 24 hours, 48 hours, and 28 days. Secondary outcomes included total blood products transfused, hospital length of stay (LOS), intensive care unit LOS, and adverse events. We conducted three pre-specified subgroup analyses to assess outcomes of patients, including (1) those who were severely injured (ISS >15), (2) those who sustained significant blood loss (≥10 units of total blood products transfused), and (3) those who sustained blunt vs penetrating trauma.

**Results:**

Propensity matching yielded two cohorts: the hospital TXA group (n = 280) and a control group (n = 280). The hospital TXA group had statistically lower mortality at 28 days (1.1% vs 5%, odds ratio [OR] [0.21], (95% confidence interval [CI], 0.06, 0.72)) and used fewer units of blood products (median = 4 units, interquartile range (IQR) = [1, 10] vs median=7 units, IQR = [2, 12.5] for the hospital TXA and control groups, respectively, (95% CI for the difference in median, -3 to -1). There were no statistically significant differences between groups with regard to 24-hour mortality (1.1% vs 1.1%, OR = 1, 95% CI, 0.20, 5.00), 48-hour mortality (1.1% vs 1.4%, OR [0.74], 95% CI, 0.17, 3.37), hospital LOS (median= 9 days, IQR = (5, 16) vs median =12 days IQR = (6, 22.5) for the hospital TXA and control groups, respectively, 95% CI for the difference in median = (−5 to 0)), and incidence of thromboembolic events (eg, deep vein thrombosis, pulmonary embolism) during hospital stay (0.7% vs 0.7% for the hospital TXA and control group, respectively, OR [1], 95% CI, 0.14 to 7.15). We conducted subgroup analyses on patients with ISS>15, patients transfused with ≥10 units of blood products, and blunt vs penetrating trauma. The results indicated lower 28-day mortality for ISS>15 (1.8% vs 7.1%, OR [0.23], 95% CI, 0.06 to 0.81) and blunt trauma (0.6% vs 6.3%, OR [0.09], 95% CI, 0.01 to 0.75); fewer units of blood products for penetrating trauma (median = 2 units, IQR = (1, 8) vs median = 8 units, IQR = (5, 15) for the hospital TXA and control groups, respectively, 95% CI for the difference in median = (−6 to −3)), and ISS>15 (median = 7 units, IQR = (2, 14) vs median = 8.5 units, IQR = (4, 16) for the hospital TXA and control groups, respectively, 95% CI for the difference in median, −3 to 0).

**Conclusion:**

The current study demonstrates a statistically significant reduction in mortality after TXA administration at 28 days, but not at 24 and 48 hours, in patients with traumatic hemorrhagic shock.

## INTRODUCTION

Trauma is the leading cause of death in individuals between the ages of one and 44 years in the United States and accounts for more than 5.8 million deaths worldwide.^1^ It is estimated that by 2020 more than one in 10 people will die from trauma-related injuries.^1^ A subset of traumatic injury deaths are a result of hemorrhagic shock that is refractory to optimal resuscitation efforts.^2^ Trauma-induced coagulopathy is present in up to 35% of patients with severe injury on arrival to the emergency department (ED).^3^ Patients with an uncorrected coagulopathy such as hyperfibrinolysis are at the greatest risk of death.^4^

Trauma-induced depletion of coagulation factors and dysregulation of the coagulation system may lead to hemodynamic instability, resulting in cardiovascular collapse. Trauma-induced coagulopathies have been associated with a significant increase in the risk of trauma-induced mortality.^3^,^5^–^8^ Although scant evidence indicates that tranexamic acid (TXA) may increase mortality in cases of fibrinolysis shutdown, patients with trauma-induced coagulopathies may benefit from the use of antifibrinolytic agents. TXA, for example, is a synthetic derivative of the amino acid lysine that exerts its antifibrinolytic effect through the reversible blockade of lysine-binding sites on plasminogen molecules.^9^

TXA administration has been studied in both the prehospital and hospital settings. Wafausade et al reported a decreased mortality after TXA administration in a prehospital setting in Germany.^10^ Similar conclusions were reported about the benefit of TXA administration in a prehospital setting.^11^ The 2010 Clinical Randomization of an Antifibrinolytic in Significant Hemorrhage 2 (CRASH-2) study was the first to report the use of TXA in the management of civilian traumatic hemorrhage in a hospital setting. CRASH-2 described a 1.5% reduction in all-cause mortality at 28 days for patients who received TXA for trauma-related injuries.^12^

Subgroup analyses of CRASH-2 in subsequent years demonstrated that the administration of TXA within three hours of injury resulted in a 2.4% decrease in death due to bleeding.^13^ The efficacy of TXA to reduce mortality was further supported by the Military Application of Tranexamic Acid in Trauma Emergency Resuscitation (MATTERS) study, a retrospective, observational study that analyzed TXA administration at a military hospital in Afghanistan.^14^ Additionally, Cole et al suggested TXA administration provided survival benefit for severely injured patients.^15^ However, Boutonnet et al studied TXA in a civilian hospital setting and reported no reduction in hospital mortality associated with TXA alone.^16^

There has been some discrepancy in current literature regarding the potential side effects of TXA, such as venous thromboembolic events (VTE), including deep vein thrombosis (DVT) and pulmonary embolism (PE). While some studies have not identified an increase in incidence of VTE associated with TXA, others have found TXA to be an independent risk factor for increased incidence of VTE.^11^,^12^,^14^,^17^–^19^ Thus, there is a need to continue to further evaluate the safety of TXA use within the hospital trauma setting.

**Population Health Research Capsule**

What do we already know about this issue?

Prior studies assessing tranexamic acid (TXA) use in civilian and military trauma resuscitation demonstrate a promising effect on mortality reduction and a limited side-effect profile.

What was the research question?

We aimed to assess the safety and efficacy of TXA in civilian adults hospitalized with traumatic hemorrhagic shock.

What was the major finding of the study?

The current study demonstrates a statistically significant reduction in mortality after TXA administration at 28 days, but not at 24 and 48 hours, in patients with traumatic hemorrhagic shock.

How does this improve population health?

Traumatic injury is a major cause of death in both developed and developing nations. TXA use represents a feasible measure toward reducing loss of life due to traumatic exsanguinating injury.

To date, there is limited evidence on the optimal timing and use of TXA in cases of traumatic hemorrhagic shock in the civilian hospital setting.^12^,^14^–^16^ Our goal was to evaluate the safety and efficacy of early TXA use in a civilian hospital setting for cases of traumatic hemorrhagic shock within a developed North American trauma system. We hypothesized that administration of TXA upon arrival to the trauma center would be associated with reduced mortality in cases of traumatic hemorrhagic shock. The primary outcome of this study was mortality measured at 24 hours, 48 hours, and 28 days. Secondary outcomes included the following: total blood products transfused during resuscitation efforts and during the hospital stay; the hospital and intensive care unit (ICU) lengths of stay (LOS); and the incidence of known adverse events associated with TXA administration including thromboembolic events (eg, DVT, PE), myocardial infarction, and neurological events (eg, stroke, seizure).

## METHODS

This civilian hospital-based study is a prospective, observational cohort study with a retrospective comparison. The current study was initiated in June 2014 at two trauma centers in Southern California: Arrowhead Regional Medical Center (Level 2 trauma center), and Loma Linda University Medical Center (Level 1 trauma center). Data collection at both trauma centers concluded in July 2018. The hospital TXA study, including administration protocols, was approved by the institutional review boards of each receiving trauma center. At each institution, TXA was approved for use in traumatic hemorrhagic shock injury within the emergency department (ED) as well as incorporated into the massive transfusion protocol and administered uniformly between centers based on the study protocol.

### Data Collection, Protocols, Outcomes

All patients ≥18-years-old who sustained blunt or penetrating trauma with signs and symptoms of hemorrhagic shock were considered for TXA treatment upon meeting inclusion criteria (Table 1). The original design of Cal-PAT (California Prehospital Antifibrinolytic Therapy) included a prehospital arm and a hospital arm.^11^ The investigators followed the same protocol to ensure consistency of inclusion/exclusion criteria. Patient selection in the hospital setting was determined by inclusion criteria upon patient arrival to the trauma center. Trauma and ED team members underwent a standardized training session on the inclusion and exclusion criteria for the study, guidelines for TXA candidate identification, protocols for TXA administration, and the medication’s side-effect profile. The choice of 120 beats per minute for heart rate (HR) for the prehospital arm was added by an agreement with the State of California EMS Agency Authority at the time of the approval of the original protocol.

TXA was delivered in two doses as per the protocol used in the CRASH-2 trial.^20^ The first dose was one gram of TXA in 100 milliliters (mL) of 0.9% normal saline infused as a bolus over 10 minutes via intravenous (IV) or intraosseous access. This first dose was administered by registered nurses as soon as feasible after the patient’s initial assessment and screening by the trauma team. Identification of study patients receiving TXA was achieved through a wristband labeled “TXA” and/or verbal communication at patient hand-off by team members. Following the completion of the first dose infusion, a second dose of TXA infusion at one gram in 100 mL of 0.9% normal saline, was administered via IV over eight hours.

**Figure 1:**
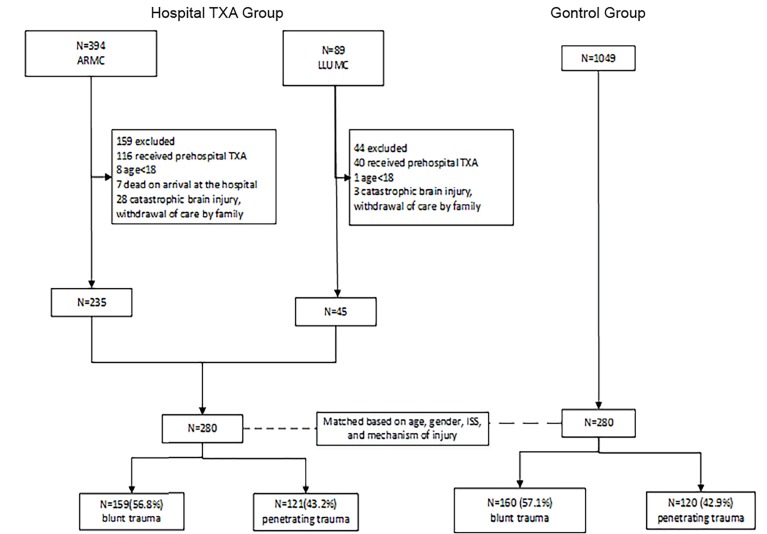
Patient flow chart for the hospital tranexamic acid (TXA) and control groups. *ISS*, Injury Severity Score; *LLUMC*, Loma Linda University Medical Center; *ARMC*, Arrowhead Regional Medical Center.

A control group was formed from patients evaluated at each respective trauma center within five years prior to the conclusion of data collection for this report. The control group patients met the same study criteria (Figure 1) and were matched to the “Hospital TXA” group patients through the use of propensity scoring based upon age, gender, injury severity score (ISS), and mechanism of injury. The biostatistician in charge of the matching process was blinded to patient outcomes to avoid bias in the matching process. There were no institutional changes in transfusion and ICU policy within the past five years in either trauma center that would have affected our outcomes. In addition, the same protocol were followed regardless of the change in trauma team members.

We abstracted data for selected subjects from the electronic health record (EHR) for each patient within each hospital. Follow-up to determine mortality outcomes after hospital discharge were abstracted from the EHR and trauma registry. In select cases, we conducted direct chart review and, in cases of missing data, study investigators contacted the patient(s) and/or the families directly to confirm survival outcomes. All patients included in this study were accounted for via hospital follow-up or direct communication.

### Statistical Analysis

We conducted all statistical analyses using SAS software for Windows, version 9.3 (SAS Institute, Cary, North Carolina). Descriptive statistics were presented as means and standard deviation or median and interquartile range (IQR) for continuous variables, along with frequencies and proportions for categorical variables. We used propensity score matching based on age, gender, ISS, and mechanism of injury to form the hospital TXA and control groups. Matching of each patient for the hospital TXA and control groups were performed within the trauma registry of each respective center involved. We conducted chi-square analyses to identify whether there were differences in mortality at 24 hours, 48 hours, and 28 days between the hospital TXA and control groups.

Independent t-tests were conducted to identify whether there were differences of continuous variables (eg, age) between the hospital TXA and control groups. We conducted Wilcoxon rank sum tests to identify whether the median of some continuous variables (eg, hospital LOS) was different between the hospital TXA and control groups. Based on the original study design, three pre-specified subgroup analyses were conducted to assess outcomes of patients, including (1) those who were severely injured (ISS >15), (2) those who sustained significant blood loss (≥10 units of total blood products transfused), and (3) those who sustained blunt vs penetrating trauma. All statistical analyses were two-sided. P-value<0.05 was considered to be statistically significant.

## RESULTS

A total of 280 patients were included in the hospital TXA group. A propensity matching process selected 280 patients from a control group (n = 1049). Thus, a total of 560 patients were included in the final analysis. Table 2 presents the overall analysis results. The hospital TXA group had statistically lower mortality at the 28-day mark (1.1% vs 5%, odds ratio [OR] [0.21], 95% confidence interval (CI), 0.06 to 0.72) and used fewer units of blood products (median =4 units, IQR = [1, 10] vs median = 7 units, IQR = [2, 12.5] for the hospital TXA and control groups, respectively, 95% CI for the difference in median = (−3 to −1)). There were no statistically significant differences between groups in regard to 24-hour mortality (1.1% vs 1.1%, OR = 1, 95% CI, 0.20, 5.00; 48-hour mortality (1.1% vs 1.4%, OR [0.74], 95% CI, 0.17, 3.37); hospital LOS (median = 9 days, IQR = (5, 16) vs median = 12 days, IQR = (6, 22.5) for the hospital TXA and control groups, respectively, 95% CI for the difference in median = (−5 to 0)), and the incidence of thromboembolic events (eg, DVT, PE) during hospital stay (0.7% vs 0.7% for the hospital TXA and control groups, respectively, OR [1], 95% CI, 0.14 to 7.15). The average time of TXA administration from injury was 100 minutes for ground transportation and 166 minutes for air transportation.

A first subgroup analysis was conducted among patients with ISS>15 (Table 3). The hospital TXA group had statistically lower mortality at 28 days (1.8% vs 7.1%, OR [0.23], 95% CI, 0.06 to 0.81). Moreover, the hospital TXA group used fewer units of blood products (median = 7 units, IQR = (2, 14) vs median = 8.5 units, IQR = (4, 16) for the hospital TXA and control groups, respectively, 95% CI for the difference in median = (−3 to 0)). We found no statistically significant differences in the 24-hour and 48-hour mortality, as well as in other secondary outcomes (Table 3).

We conducted a second subgroup analysis among patients who were transfused ≥10 units of total blood product (Table 4). There were no statistically significant differences in 24-hour, 48-hour, and 28-day mortality, and all secondary outcomes (Table 4). A final subgroup analysis was conducted based on blunt vs penetrating trauma (Table 5). Among patients who sustained blunt trauma, the hospital TXA group had statistically lower mortality at 28 days (0.6% vs 6.3%, OR [0.09], 95% CI, 0.01 to 0.75). There was no statistically significant difference in the 24-hour and 48-hour mortality, and secondary outcomes (Table 5).

Among patients who sustained penetrating trauma, the hospital TXA group used fewer units of blood products (median = 2 units, IQR = (1, 8) vs median = 8 units, IQR = (5, 15) for the hospital TXA and control groups, respectively, 95% CI for the difference in median = (−6 to −3)), and had a shorter hospital LOS (median = 6 days, IQR = (2.5, 14.5) vs median = 11 days, IQR = (7, 21.5) for the hospital TXA and control groups, respectively, 95% CI for the difference in median = (−7 to −1)). There was no statistically significant difference in the 24-hour, 48-hour, and 28-day mortality, and other secondary outcomes (Table 5).

## DISCUSSION

This study completed in July 2018, marks one of the first large-scale prospective studies assessing the effects of TXA administration when used for traumatic hemorrhagic shock in a civilian hospital setting within a developed North American trauma system. Hospital TXA administration was associated with a statistically lower 28-day mortality. Secondary outcomes in this study also demonstrated a statistically significant decrease in hospital LOS.

The current study suggests that TXA may be more effective in patients who are more severely injured and require more units of blood products. The benefit of TXA particularly among the most severely injured trauma patients was consistent with multiple other studies including CRASH-2 and MATTERS.^12^,^14^ Despite the fact that the TXA groups were more severely injured, both studies identified a decrease in mortality.^12^,^14^ Additionally, patients requiring a massive blood transfusion benefited more from the TXA administration.^14^ Both benefits were identified despite the inability to quantify the degree of fibrinolysis prior to TXA treatment.

TXA has been hypothesized to exert its beneficial effect on trauma patients via its antifibrinolytic properties. Specifically, TXA has been thought to reduce mortality by preventing exsanguination on the day of injury.^21^ After significant trauma, coagulopathies may begin almost immediately and can rapidly progress to life-threatening scenarios.^6^–^8^ These coagulopathies have been postulated to be driven in part by excessive activation of the thrombomodulin-protein C pathway.^22^ Following tissue hypoperfusion in the setting of traumatic injury, protein C is activated.^3^ This subsequent rise in activated protein C leads to proteolytic cleavage and inactivation of procoagulant factors V and VIII.

In addition, activated protein C neutralizes plasminogen activator inhibitor-1 causing increased concentrations of tissue plasminogen activator and further progression of fibrinolysis.^23^ These mechanisms combine and can lead to acute traumatic coagulopathies.^22^ Research has demonstrated that high levels of activated protein C on admission in trauma patients have been associated with increased mortality, longer hospital stay, and increased transfusion requirements.^22^ Although not specifically measured in our study, the role of activated protein C in coagulopathies may show why TXA’s ability to inhibit the excess plasminogen is crucial in preventing mortality.

We observed a statistically significant decrease in 28-day mortality, suggesting that TXA may exert an effect beyond the limitation of blood loss and treatment of hyperfibrinolysis. This may be due to the long-term effects of limiting profound hypoperfusion in the setting of trauma and the long-term benefits in controlling bleeding with TXA therapy. The conversion of plasminogen to plasmin in the clotting pathway exacerbates and leads to overactivation of the inflammatory response.^24^ Plasmin has been shown to have a direct effect on macrophages, leading to the transcription of the proinflammatory cytokines tumor necrosis factor-alpha and interleukin-6.^25^ Excess plasmin can cause detachment of endothelial cells leading to apoptosis and release of radical oxygen species.^24^ Aside from the proinflammatory effects, plasmin has also been known to cause platelet hypofunction.^24^ TXA’s inhibitory effect on the conversion of plasminogen to plasmin may contribute to its anti-inflammatory properties leading to the extended benefits observed in our study. The exact mechanism is likely multi-factorial and needs to be more clearly elucidated.

The efficacy of TXA when used during fibrinolysis and hyperfibrinolysis is controversial. Recent studies demonstrate that TXA may be associated with an increased risk of fibrinolytic shutdown when monitored via thromboelastography (TEG).^26^ In another study within a civilian hospital setting, patients receiving TXA required more total blood products and had a statistically significant increase in mortality.^27^ However, this study has limitations, given that it is a retrospective study and includes older patients with higher injury severity and hypotension compared to patients in other studies.^12^,^27^

The incidence of VTE associated with TXA administration in a trauma setting has also been controversial. Johnston et al conducted a retrospective, follow-up study to MATTERs to re-examine TXA use within the military hospital setting.^17^ They reported a higher incidence of VTE in patients receiving TXA and found that use of TXA was an independent risk factor for VTE with an overall rate of 15.6% VTE.^17^ The prevention of clot dissolution via TXA inhibition of plasmin may heighten risks of VTE by promoting thrombus.^18^ A civilian study performed by Myers et al reported a 7.4% and 15.5% incidence rate of VTE for the control and TXA groups, respectively.^19^ However, the reported incidence rate of VTE by Myers and Johnston was much higher than other reported VTE incidence rates ranging from 0.36% to 1.8%.^11^,^20^,^28^

The current study suggests an incidence rate of 0.7% for VTE among patients who received TXA, which is within range of previously reported incidence rates.^11^,^20^,^28^ The two studies that reported TXA administration as an independent risk factor for VTE had several significant limitations including the following: retrospective data collection; possible patient selection bias; small sample size; population differences between control groups and TXA groups; VTE surveillance bias; and variation in trauma settings.^17^,^18^ Future prospective research is warranted to examine the incidence of VTE among adult trauma patients.

## LIMITATIONS

First, this study was limited by design. The prospective, non-randomized cohort design did not allow TXA to be administered in a blinded fashion. Providers and physicians were aware of TXA administration, which may have affected the level of care provided and assessments of outcome. However, given that the primary outcome was mortality, this impact was likely minimal. Second, we acknowledge an inability to account for certain potential confounding factors. This includes the variability of total transport time to the ED, which contributed to variability of initial TXA administration time. Additionally, despite following the same study protocol, patients were included from two trauma centers in the same geographic area that may follow slightly different institutional policy and procedure. To reduce the impact of these differences on patient outcomes, control patients were matched by trauma center. These factors in addition to minimal inherent differences between the TXA and control groups may limit the generalizability of these results.

Lastly, we did not integrate the use of TEG into this study; thus, we were unable to assess the combined role of TEG and TXA regarding patient outcomes. Debate continues regarding whether TEG can accurately predict the need and use of TXA.^21^,^29^ TEG is not routinely available in many trauma centers; however, those centers have continued including TXA administration in their current trauma resuscitation standards. Further investigation into the combined use of both TXA administration and TEG is warranted.^14^

## CONCLUSION

The current study demonstrates a statistically significant reduction in mortality after TXA administration at 28 days, but not at 24 and 48 hours, in patients with traumatic hemorrhagic shock. Future prospective research is warranted to further evaluate the benefits and side effects of TXA use among adult civilian trauma patients on a larger scale.

## Figures and Tables

**Table 1:** Patients Inclusion and exclusion criteria provided to clinicians at receiving trauma centers.

Inclusion criteria	Exclusion criteria
The hospital use of TXA should be considered for all trauma patients that meet any of the following criteria: Blunt or penetrating trauma with signs and symptoms of hemorrhagic shock within three hours of injury. ○ Systolic blood pressure of less than 90 mmHg upon arrival to designated trauma centers. ○ Heart rate >120. ○ Estimated blood loss of 500 milliliters ○ Bleeding not controlled by direct pressure or tourniquet. Major amputation of any extremity above the wrists and above the ankles.	Any patient <18 years of age. Any patient more than three hours post-injury. Any patient with an active thromboembolic event (within the last 24 hours) – ie, active stroke, myocardial infarction or pulmonary embolism. Any patient with a hypersensitivity or anaphylactic reaction to TXA. Any patient that received prehospital TXA. Traumatic arrest with more than five minutes of cardiopulmonary resuscitation without return of vital signs. Penetrating cranial injury. Traumatic brain injury with brain matter exposed. Isolated drowning or hanging victims. Documented cervical cord injury with motor deficits.

*TXA*, tranexamic acid, *mmHG*, millimeters of mercury.

**Table 2:** Comparison of outcomes and factors between hospital tranexamic acid (TXA) and control groups.

	Hospital TXA group (n = 280)	Control group (n = 280)	Statistic with 95% CI
Outcomes			
Mortality at 24 hours	3 (1.1%)	3 (1.1%)	1 (0.20, 5.00)†
Mortality at 48 hours	3 (1.1%)	4 (1.4%)	0.74 (0.17, 3.37)†
Mortality at 28 days	3 (1.1%)	14 (5%)	0.21 (0.06, 0.72)†
Total blood product, units, median (Q1, Q3)	4 (1, 10)	7 (2, 12.5)	−2 (−3, −1)‡
Hospital LOS, days, median (Q1, Q3)	9 (5, 16)	12 (6, 22.5)	−2 (−5, 0)‡
ICU LOS, days, median (Q1, Q3)	4 (3, 8)	4 (2, 10)	0 (−1, 1)‡
Adverse event during hospital stay			1 (0.14, 7.15)†
VTE	2 (0.7%)	2 (0.7%)	
None	278 (99.3%)	278 (99.3%)	
Factors			
Blunt trauma percentage	159 (56.8%)	160 (57.1%)	0.99 (0.71, 1.38)†
Male percentage	236 (84.3%)	241 (86.1%)	0.87 (0.54, 1.38)†
Age, years, mean ± SD	38.89 ± 15.98	37.91 ± 18.15	0.98 (−1.86, 3.82)*
SBP, mmHg, mean ± SD	99.32 ± 17.84	102.32 ± 23.27	−3.00 (−6.51, 0.50)*
Discharge ISS, median (Q1, Q3)	17 (10, 26)	17 (12, 26)	0 (0, 2)‡
GCS, median (Q1, Q3)	15 (11, 15)	15 (14, 15)	0 (0,0)‡

† Values were presented as the odds ratio (use the control group as the reference) and the corresponding 95% confidence interval. Chi-square tests were conducted to assess the statistical significance. If the 95% confidence interval contains 1, then there was not statistically significant difference between the two groups.

‡ Values were presented as the median and IQR for the difference between the two groups (defined as the hospital TXA group less the control group). Wilcoxon rank sum tests were conducted to assess the statistical significance. If the 95% confidence interval contains 0, then there was not statistically significant difference between the two groups.

* Values were presented as the means and 95% corresponding confidence interval for the difference between the two groups (defined as the hospital TXA group less the control group). An independent t-tests were conducted to assess the statistical significance. If the 95% confidence interval contains 0, then there was not statistically significant difference between the two groups.

*TXA*, tranexamic acid; *CI;* confidence interval; *LOS*, length of stay; *ICU*, intensive care unit; *ISS*, Injury Severity Score; *Q1*, 25th percentile; *Q3*, 75th percentile; *SBP*, systolic blood pressure; *SD*, standard deviation; *GCS*, Glasgow Coma Scale; *VTE*, venous thromboembolic events.

**Table 3:** Subgroup analysis: comparison of outcomes and factors between hospital tranexamic acid (TXA) and control groups among patients with Injury Severity Score >15.

	ISS>15 (n = 337)		
	Hospital TXA group (n=167)	Control group (n=170)	Statistic with 95% CI
Outcomes			
Mortality at 24 hours	3 (1.8%)	3 (1.8%)	1.01(0.20, 5.12)^†^
Mortality at 48 hours	3 (1.8%)	4 (2.4%)	0.76 (0.17, 3.44)^†^
Mortality at 28 days	3 (1.8%)	12 (7.1%)	0.23 (0.06, 0.81)^†^
Total Blood Product, units, median (Q1, Q3)	7 (2, 14)	8.5 (4, 16)	−2 (−3, 0)^‡^
Hospital LOS, days, median (Q1, Q3)	13 (7, 17)	14 (7, 27)	−2 (−6, 2)^‡^
ICU LOS, days, median (Q1, Q3)	5 (3, 10)	5 (3, 13)	0 (−1, 1)^‡^
Factors			
Blunt trauma percentage	116 (69.5%)	98 (57.7%)	1.67 (1.07, 2.62)^†^
Male percentage	132 (79%)	146 (85.9%)	0.62 (0.35, 1.10)^†^
Age, years, mean ± SD	39.23 ± 16.44	35.79 ± 16.84	3.43 (−0.13, 7.00)^*^
SBP, mmHg, mean ± SD	98.66 ± 17.8	101.26 ± 23.77	−2.60 (−7.18, 1.99)^*^
Discharge ISS, median (Q1, Q3)	24 (17, 29)	24 (17, 29)	0 (−1, 1) ^‡^
GCS, median (Q1, Q3)	14 (8, 15)	15 (14, 15)	0 (0, 0) ^‡^

**Table 4:** Subgroup analysis: comparison of outcomes and factors between hospital tranexamic acid (TXA) and control groups among patients who were transfused ≥10 units of blood product.

	Blood product ≥ 10 Units (n=176)		
	Hospital TXA group (n=76)	Control group (n=100)	Statistic with 95% CI
Outcomes			
Mortality at 24 hours	3 (4%)	3 (3%)	1.33 (0.26, 6.77)†
Mortality at 48 hours	3 (4%)	4 (4%)	0.99 (0.21, 4.54)†
Mortality at 28 days	3 (4%)	11 (11%)	0.34 (0.09, 1.24)†
Total Blood Product, units, median (Q1, Q3)	15.5 (12, 23.5)	16 (12, 25)	−1 (−3, 1)‡
Hospital LOS, days, median (Q1, Q3)	16 (8, 23)	16 (7, 28.5)	−1 (−8, 6)‡
ICU LOS, days, median (Q1, Q3)	6 (3, 13)	6 (4, 13)	0 (−2, 2)‡
Factors			
Blunt trauma percentage	50 (65.8%)	54 (54%)	1.64 (0.88, 3.03)†
Male percentage	58 (76.3%)	88 (88%)	0.44 (0.20, 0.98)†
Age, years, mean ± SD	41.04 ± 15.78	35.41 ± 16.61	5.63 (0.75, 10.51)*
SBP, mmHg, mean ± SD	98.24 ± 16.94	107.21 ± 23.39	−8.98 (−15.35, −2.60)*
Discharge ISS, median (Q1, Q3)	22 (17, 29)	22 (16, 29)	0 (−2, 4)‡
GCS, median (Q1, Q3)	14 (7, 15)	14 (13, 15)	0 (−1, 0)‡

† Values were presented as the odds ratio (use the control group as the reference) and the corresponding 95% confidence interval. Chi-square tests were conducted to assess the statistical significance. If the 95% confidence interval contains 1, then there was not statistically significant difference between the two groups.

‡ Values were presented as the median and IQR for the difference between the two groups (defined as the hospital TXA group less the control group). Wilcoxon rank sum tests were conducted to assess the statistical significance. If the 95% confidence interval contains 0, then there was not statistically significant difference between the two groups.

* Values were presented as the means and 95% corresponding confidence interval for the difference between the two groups (defined as the hospital TXA group less the control group). An independent t-tests were conducted to assess the statistical significance. If the 95% confidence interval contains 0, then there was not statistically significant difference between the two groups.

*TXA*, tranexamic acid; *CI;* confidence interval; *LOS*, length of stay; *ICU*, intensive care unit; *Q1*, 25th percentile; *Q3*, 75th percentile; *SBP*, systolic blood pressure; *mmHg*, millimeters of mercury; *SD*, standard deviation; *GCS*, Glasgow Coma Scale; *ISS*, Injury Severity Score.

**Table 5:** Subgroup analysis: comparison of outcomes and factors between hospital tranexamic acid (TXA) and control groups for blunt vs penetrating trauma.

	Blunt trauma (n = 319)			Penetrating trauma (n=241)		
	Hospital TXA group (n = 159)	Control group (n = 160)	Statistic with 95% CI^**^	Hospital TXA group (n = 121)	Control group (n = 120)	Statistic with 95% CI
Outcomes						
Mortality at 24 hours	1 (0.6%)	1 (0.6%)	1.00 (0.06, 16.2)†	2 (1.7%)	2 (1.7%)	0.99 (0.14, 7.16)†
Mortality at 48 hours	1 (0.6%)	2 (1.3%)	0.5 (0.04, 5.57)†	2 (1.7%)	2 (1.7%)	0.99 (0.14, 7.16)†
Mortality at 28 days	1 (0.6%)	10 (6.3%)	0.09 (0.01, 0.75)†	2 (1.7%)	4 (3.3%)	0.49 (0.09, 2.71)†
Total Blood Product, units, median (Q1, Q3)	5 (2, 11)	5 (2, 11)	0 (−1, 1)‡	2 (1, 8)	8 (5, 15)	−5 (−6, −3)‡
Hospital LOS, days, median (Q1, Q3)	13 (7, 16)	14 (5, 23)	0 (−6, 4)‡	6 (2.5, 14.5)	11 (7, 21.5)	−4 (−7, −1)‡
ICU LOS, days, median (Q1, Q3)	5 (3, 10)	5 (2, 10)	1 (0, 2)‡	3 (1, 5)	4 (2, 9)	−1 (−2, 0)‡
Factors						
Male percentage	122 (76.7%)	126 (78.8%)	0.89 (0.52, 1.51)†	114 (94.2%)	115 (95.8%)	0.71 (0.22, 2.30)†
Age, years, mean ± SD	42.55 ± 17.34	44.34 ± 19.09	−1.80 (−5.81, 2.22)*	34.08 ± 12.53	29.33 ± 12.46	4.76 (1.59, 7.93)*
SBP, mmHg, mean ± SD	99.25 ± 17.06	100.23 ± 21.4	−0.98 (−5.28, 3.33)*	99.4 ± 18.9	105.46 ± 25.61	−6.06 (−11.94, −0.17)*
Discharge ISS, median (Q1, Q3)	22 (14, 27)	17 (12, 26)	1 (0, 4)‡	14 (9, 19)	17 (11, 25)	3 (1,5)‡
GCS, median (Q1, Q3)	15 (10, 15)	14.5 (13.5, 15)	0 (0, 0)‡	15 (13, 15)	15 (14, 15)	0 (0,0)‡

† Values were presented as the odds ratio (use the control group as the reference) and the corresponding 95% confidence interval. Chi-square tests were conducted to assess the statistical significance. If the 95% confidence interval contains 1, then there was not statistically significant difference between the two groups.

‡ Values were presented as the median and IQR for the difference between the two groups (defined as the hospital TXA group less the control group). Wilcoxon rank sum tests were conducted to assess the statistical significance. If the 95% confidence interval contains 0, then there was not statistically significant difference between the two groups.

* Values were presented as the means and 95% corresponding confidence interval for the difference between the two groups (defined as the hospital TXA group less the control group). An independent t-tests were conducted to assess the statistical significance. If the 95% confidence interval contains 0, then there was not statistically significant difference between the two groups.

*TXA*, tranexamic acid; *CI;* confidence interval; *LOS*, length of stay; *ICU*, intensive care unit; *Q1*, 25th percentile; *Q3*, 75th percentile; *SBP*, systolic blood pressure; *mmHg*, millimeters of mercury; *SD*, standard deviation; *GCS*, Glasgow Coma Scale; *ISS*, Injury Severity Score.
